# Laser capture proteomics reveals new candidates for sperm-interacting proteins in the bovine oviduct epithelium

**DOI:** 10.1530/REP-24-0390

**Published:** 2025-07-17

**Authors:** Coline Mahé, Aleksandra Maria Mowinska, Elke Albrecht, Karine Reynaud, Régis Lavigne, Emmanuelle Com, Charles Pineau, Pascal Mermillod, Marie Saint-Dizier, Jennifer Schoen

**Affiliations:** ^1^ INRAE, CNRS, Université de Tours, PRC, Nouzilly, France; ^2^ Research Institute for Farm Animal Biology (FBN), Dummerstorf, Germany; ^3^ Department of Reproduction Biology, Leibniz Institute for Zoo and Wildlife Research (IZW), Berlin, Germany; ^4^ Univ Rennes, Inserm, EHESP, Irset (Institut de recherche en santé, environnement et travail) – UMR_S 1085, Rennes, France; ^5^ Univ Rennes, CNRS, Inserm, Biosit UAR 3480 US_S 018, Protim core facility, Rennes, France; ^6^ Institute of Biotechnology, Technische Universität Berlin, Berlin, Germany

**Keywords:** proteome, sperm, laser microdissection, oviduct, bovine

## Abstract

**In brief:**

The molecular interactions between spermatozoa and the oviduct epithelium that are involved in capacitation and sperm availability during fertilization still remain largely unknown in many species. This study provides comprehensive proteomes of the apical and basal part of the bovine oviduct epithelium (isthmus and ampulla, pre- and post-ovulatory) and presents new protein candidates for sperm binding in the bovine functional sperm reservoir.

**Abstract:**

The proximal region of the oviduct (isthmus) serves as a sperm reservoir in many mammalian species. This reservoir is composed of epithelial cells that bind sperm primarily to their apical cilia and ensure the survival of high-quality sperm until ovulation. The species-specific molecular interactions between spermatozoa and the oviduct epithelium, however, are still poorly understood. The aim of this study is to identify new candidates for sperm-interacting proteins in the bovine reservoir using laser capture proteomics. Ipsilateral oviducts were collected from cyclic cows at pre- and post-ovulatory stages. Isthmus and ampulla tissue samples were fixed, paraffin-embedded, sectioned and subjected to laser microdissection to establish pools of microsamples separating the apical part of the epithelium, containing potential sperm-interacting proteins, from the basal part, containing proteins not currently located or secreted on the cell surface. Proteins were analyzed by nanoLC-MS/MS, and differentially abundant proteins (DAPs) were identified using t-tests. A total of 505 and 512 proteins could be identified in apical and basal microsamples, respectively. Only 8/141 regional and 1/79 cycle stage-related DAPs were shared between both epithelium parts, indicating a selective regulation of protein expression. Of the apical proteins, 19 were predicted to be candidates for sperm interactions, including annexins (ANXA) 1, 2, 5 and 8; oviduct glycoprotein 1 (OVGP1); voltage-dependent anion-selective channel proteins (VDAC) 1 and 2; apolipoprotein A1 (APOA1). This work provides the first proteomic characterization of microdissected cellular compartments of the bovine oviduct epithelium and presents new candidates for improving sperm quality in assisted reproductive technologies.

## Introduction

The oviduct provides optimal conditions for key reproductive events such as gamete maturation and selection, fertilization and early embryonic development (Avilés *et al.* 2015, Kölle 2022). It is divided into the isthmus, acting as a sperm reservoir and supporting early embryo development, and the ampulla, the site of oocyte maturation and fertilization (Coy *et al.* 2012). The oviduct lumen is lined by a simple, columnar, ciliated epithelium composed of non-ciliated/secretory and ciliated cells (Ito *et al.* 2023). Oviduct epithelial cells (OECs) exhibit pronounced apical-basal polarity, i.e. the apical (luminal) cell compartment differs morphologically and functionally from the basal compartment. The cell bodies reside on the basal membrane, where they receive signals and nutrition from the underlying maternal tissue. The apical surface of the oviduct epithelium, in contrast, comes into contact with gametes and early embryos and forms a specific, dynamic microenvironment for their maturation and development (Saint-Dizier *et al.* 2020*a*). The motile cilia on the cell surface generate a fluid flow that guides the oocyte and embryo toward the isthmus and uterus. Spermatozoa, however, follow this fluid flow upstream toward the ampulla by means of rheotaxis (Li & Winuthayanon 2017). Cilia are also in direct contact with spermatozoa, forming a sperm reservoir located in the proximal part of the isthmus and maintaining sperm fertilizing ability up to the time of ovulation (Sostaric *et al.* 2008, Kölle 2022). Sperm binding to OECs is mediated by membrane proteins and carbohydrate moieties associated with membrane phospholipids and glycoproteins on OECs (Talevi & Gualtieri 2010, Miller 2018, Saint-Dizier *et al.* 2020*b*). To date, only nine proteins have been identified as potential receptors for sperm on bovine OECs: annexins (ANXA) A1, 2, 4 and 5 (Ignotz *et al.* 2007), heat shock proteins (HSP) A5, 60 and A8 (Boilard *et al.* 2004), fibronectin (FN1; Osycka-Salut *et al.* 2017) and cadherin-1 (CDH1; Caballero *et al.* 2014). On bovine sperm, the seminal plasma proteins PDC-109 (BSP1), A3 (BSP3) and BSP-30kD (BSP5; Gwathmey *et al.* 2006), and the β-defensin 126 (DEFB126), secreted by the corpus epididymidis, were identified as ligands for OECs (Lyons *et al.* 2018). So far, in cattle, only two ligand–receptor pairs have been identified: integrin α5β1 binding to fibronectin (Osycka-Salut *et al.* 2017) and BSPs interacting with annexins (Ignotz *et al.* 2007).

In the present study, we aimed to discover additional protein candidates potentially involved in sperm–oviduct interactions, specifically in the establishment and functions of the bovine sperm reservoir. Proteins relevant to the functions of the sperm reservoir are expected to be present at the surface of the oviduct epithelium in the isthmus region of the oviduct. Furthermore, these proteins should be differentially abundant before (sperm binding to the reservoir) and after ovulation (sperm release). To identify such proteins, we a) obtained samples of the OEC surface and the underlying cell bodies separately by laser capture microdissection, b) characterized the proteins present in these microsamples, and c) compared the apical and basal OEC proteomes from isthmus and ampulla before and after ovulation.

## Materials and methods

**Figure 1:**
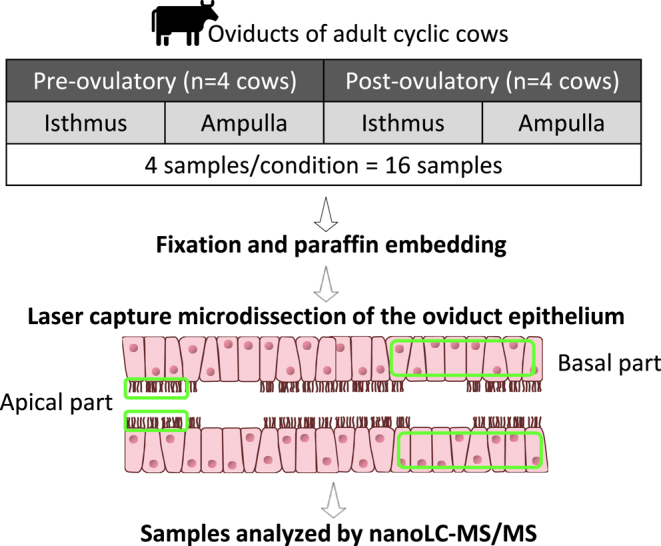
Experimental design of the study. Ipsilateral oviducts from adult cows in pre- and postovulatory cycle stages were collected from a local slaughterhouse (four cows per cycle stage). Isthmus and ampulla were separated and samples from both regions were fixed in paraformaldehyde and embedded in paraffin. Microsamples of the apical and basal part of the isthmic and ampullary epithelium were generated by laser capture microdissection. Proteins were subsequently analyzed by nano liquid chromatography coupled with tandem mass spectrometry (nanoLC-MS/MS).

The experimental design is shown in Fig. 1.

### Collection of bovine oviducts

Oviducts from adult cyclic cows were collected at a commercial slaughterhouse (Vendôme, France) as previously described (Mahé *et al.* 2023). Briefly, oviducts and ovaries were immediately placed on ice, transported to the laboratory, and processed within 2 h after the death of the animal. Ipsilateral oviducts were classified according to the morphology of the ovaries (Ireland *et al.* 1980) as either pre-ovulatory (pre-ov; presence of a pre-ovulatory antral follicle and a *corpus albicans*) or post-ovulatory (post-ov; presence of an early *corpus hemorrhagicum*). A total of 16 samples were collected from four cows at pre-ov and four cows at post-ov stage. The oviducts were isolated from the surrounding tissues, blood vessels and ovaries, and then flushed with 500 μL of cold (4°C) phosphate-buffered saline (PBS) (Mahé *et al.* 2022). After removal of the central part of the oviduct (3 cm of the isthmus-ampulla junction), four pieces (3–4 mm) of the isthmus and ampulla were placed in 10 mL of 4% phosphate-buffered paraformaldehyde for 6 h. Preliminary tests have shown that this relatively short fixation time preserves the structure of the sample while still allowing the extraction and identification of proteins by nanoLC-MS/MS (data not shown). The pieces were washed in 10 mL of PBS and stored at 4°C until further processing. Within 7 days, the pieces were transferred to HISTOSETTE^®^ Biopsy Processing/Embedding Cassettes, dehydrated in a tissue processor (TP1020, Leica, Germany), and embedded in paraffin.

### Microdissection of the bovine oviduct epithelium

**Figure 2:**
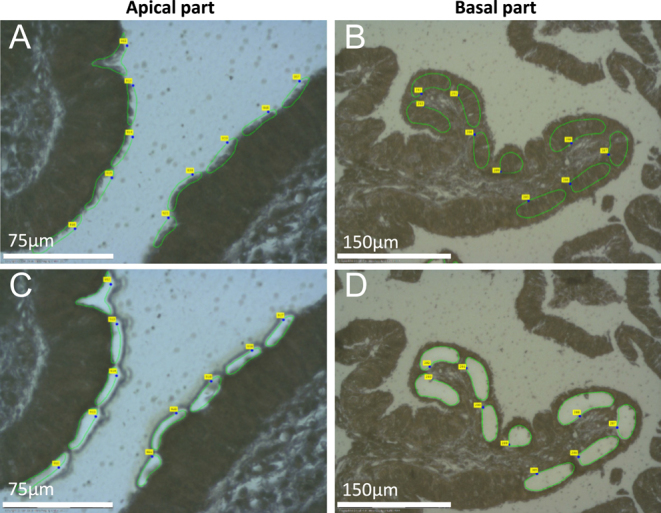
Microsampling of paraffin-embedded oviduct tissue. Representative microscopic images of the bovine oviduct epithelium before and after laser capture microdissection. Selected sample areas (regions of interest) capturing the apical (A/C) or basal (B/D) part of the epithelium are marked by green lines. Scale bars represent 75 μm (A/C) and 150 μm (B/D), respectively. Small yellow squares indicate the sample count.

Before use, membrane slides (415190-9041-000, Carl Zeiss, Germany) were exposed to ultraviolet light for 30 min. For each isthmus and ampulla, 6 μm sections were placed on membrane slides overnight at room temperature. The sections were then deparaffinized at 60°C for 45 min and immersed twice for 10 min in RotiClear^®^ clearing agent (Carl Roth, Germany). After overnight drying at room temperature, the apical part (containing cilia, apical cell membranes, apical protrusions and oviductal fluid) and the basal part (containing cytoplasm, lateral cell membranes and nuclei) of the oviduct epithelium were isolated by laser capture microdissection on a PALM MicroBeam (Carl Zeiss, Germany). Regions of interest were defined for the apical or basal epithelium parts (Fig. 2A and B), and cut using the 40× or the 20× objective, respectively (Fig. 2C and D). Microdissected tissue pieces were collected in pools of 565 ± 86 pieces (apical parts) and 232 ± 20 pieces (basal parts) in Adhesive Caps (415190-9181-000, Carl Zeiss, Germany) and stored at −80°C until further use. Samples were rehydrated in an isopropanol dilution series: 100 μL of each dilution (2× 100, 70, 50, 0% isopropanol in ultrapure water) was successively added to the tissue pieces. Each time, the tube was gently inverted and incubated for 3 min at room temperature. The tube was then centrifuged at 16,000 ***g*** for 3 min before removing the supernatant. The samples were dried by leaving the cap of the tube open and stored at −80°C until proteomic analysis.

### Nano-liquid chromatography coupled with tandem mass spectrometry (nanoLC-MS/MS)

For proteomic analysis, microdissected apical and basal samples were prepared using the PreOmics iST kit (PreOmics GmbH, Germany) according to the manufacturer’s instructions, with minor modifications to adapt the protocol to the small amount of material from formalin-fixed, paraffin-embedded tissues. Briefly, proteins were lysed, denatured, reduced and alkylated at 95°C for 60 min, followed by Trypsin/LysC digestion at 37°C for 3 h. Salts, contaminants and other detergents were removed from the samples using the PreOmics spin cartridge, according to the manufacturer’s instructions. A total of 32 samples, 16 from apical and 16 from basal epithelium parts, were then analyzed individually by nano-liquid chromatography and tandem mass spectrometry in the diaPASEF mode. Two-thirds of each sample were separated on a 75 μm × 250 mm IonOpticks Aurora 3 C18 column (Ion Opticks Pty Ltd, Australia). A reverse-phase buffer gradient (buffer A: 0.1% formic acid, 2% acetonitrile, 97.9% H_2_O; buffer B: 0.1% formic acid, 99.9% acetonitrile) was run on a nanoElute UHPLC System (Bruker Daltonik GmbH, Germany) at a flow rate of 250 nL/min at 50°C, controlled by HyStar software (v6.0.30.0, Bruker Daltonik GmbH, Germany). The liquid chromatography (LC) run lasted for 80 min. A starting concentration of 2% buffer B, increasing to 13% over the first 42 min, was first used, followed by buffer B increases to 20% at 65 min, 30% at 70 min, 85% at 75 min, and finally 85% for 5 min to wash the column. The temperature of the ion transfer capillary was set at 180°C. Ions were accumulated for 100 ms, and mobility separation was achieved by ramping the entrance potential from −160 to −20 V within 114 ms. MS and MS/MS mass spectra were acquired with average resolutions of 50.000 FWHM full width at half maximum (mass range: 100–1,700 *m/z*), respectively. A diaPASEF method with an adapted instrument firmware was developed to perform data-independent isolation of data from multiple 25 *m/z* wide precursor windows, also called segments, in a single TIMS separation (107.5 ms). A method with two boxes per segment in each 107.5 ms diaPASEF scan was used, i.e. a total of 32 segments and 64 boxes, of which 16 of these scans perfectly cover the diagonal area of doubly and triply charged peptides in the *m*/*z* and ion mobility output range. MS and MS/MS data were collected over the *m*/*z* range 100–1,700 and over the mobility range from 1/K0 = 0.6 to 1/K0 = 1.6 Vs cm^−2^. During each data collection, each TIMS cycle was 1.25 s long and consisted of one MS and 22 cycles of diaPASEF MS/MS segments, comprising two, three or four boxes to cover a total of 64 boxes defined in the acquisition method. The collision energy was increased linearly with mobility from 68 eV at 1/K0 = 1.6 Vs cm^−2^ to 25 eV at 1/K0 = 0.6 Vs cm^−2^. TIMS, MS operation and diaPASEF were controlled and synchronized by the OtofControl 6.2.5 software (Bruker Daltonik). Ion mobility-resolved mass spectra, nested ion mobility versus *m*/*z* distributions, and fragment ion intensity sums were extracted from the raw data file using DataAnalysis 6.0 (Bruker Daltonik GmbH, Germany). The signal-to-noise ratio (S/N) was increased by summing the individual TIMS scans. Mobility peak positions and half-peak widths were determined from the extracted ion mobilograms (EIM, ±0.05 Da) using the peak detection algorithm implemented in the DataAnalysis software. Feature detection was also performed using DataAnalysis 6.0 software and stored at the raw data level (.d). For the resource library, all DIA raw files were analyzed in Spectronaut software version 18 (Biognosys, Switzerland) using the integrated Pulsar search engine and a default search scheme to generate a global spectral library. The calibration search was dynamic, and the MS1 and MS2 correction factor was 1. The data were searched against the UniProt *Bos taurus* database (23,241 sequences, downloaded in June, 2023), with trypsin/P as the protease and up to one missed cleavage. To account for post-translational modifications and chemical labeling settings, carbamidomethylation of cysteine residues was defined as a fixed modification, and methionine oxidation and N-terminal protein acetylation were defined as variable modifications. A false discovery rate (FDR) of less than 1% was ensured at the precursor, peptide and protein levels. Before library-based analysis of the DIA data, the raw files were converted to htrms files using the htrms converter (Biognosys). MS1 and MS2 data were centroided during conversion. All other parameters were set to default. The htrms files from the apical and basal microsamples were then analyzed separately with Spectronaut against the previously generated libraries and default settings, allowing the quantification of precursors, peptides and proteins. The results were filtered by a 1% FDR at the precursor, peptide and protein levels using a target-decoy approach, corresponding to a *Q* value ≤0.01 (Bruderer *et al.* 2017). The default Biognosys factory normal reports were exported from the Spectronaut software for the apical and basal epithelium parts, respectively. Raw data are available in the ProteomeXchange Consortium via the PRIDE repository (Perez-Riverol *et al.* 2022) with the dataset identifier PXD055890 (username: reviewer_pxd055890@ebi.ac.uk; password: NuX1WbK9H12A).

### Protein identification, validation and quantification

Protein groups identified with at least two unique peptides were retained and quantified using the iq package in RStudio software v2023.06.2 (Pham *et al.* 2020) and the Spectronaut reports as input. The method used for protein quantification was precursor ion intensity-based label-free quantification by delayed normalization and maximum peptide ratio extraction (Cox *et al.* 2014). Quantification data were then median normalized by subtracting each quantitative value in each sample from the sample median. To obtain a list of unique proteins, sequences of proteins from each protein group were compared using the online blastp tool (https://blast.ncbi.nlm.nih.gov/Blast.cgi?PAGE=Proteins). If protein sequences had at least 90% homology, the protein annotated and verified by UniProtKB or with an existing gene symbol was considered to represent the protein group.

### Prediction of subcellular localization of proteins

To identify potential ciliary proteins, the lists of gene symbols of all identified proteins were compared with the lists of proteins previously identified in human airway cilia (Blackburn *et al.* 2017) and the Human Protein Atlas entries for ‘cilia’ or ‘cilium’ (Uhlén *et al.* 2015) (see Supplementary Table S1 (see section on Supplementary materials given at the end of the article) for the lists of proteins). To predict the subcellular localization of proteins (intracellular, transmembrane or secreted), the FASTA sequences were retrieved with the ID mapping option in UniProt (https://www.uniprot.org/id-mapping) and used as input in the Outcyte 1.0 online tool (http://www.outcyte.com/) (Zhao *et al.* 2019). Transmembrane localization of proteins was predicted using the TMHMM 2.0 tool (https://services.healthtech.dtu.dk/services/TMHMM-2.0/) (Krogh *et al.* 2001).

### Statistical analysis

All statistical analyses were performed using the RStudio software and the binary logarithm of quantitative values. Principal component analysis (PCA) of all proteins was performed using the FactoMineR, ggplot2 and missMDA missing value imputation packages (Harris *et al.* 2023). Differences in protein abundance between regions at a given stage were analyzed by Student’s paired *t*-tests, and between stages in a given region by Student’s unpaired *t*-tests. A protein was considered to be differentially abundant between regions or stages with a *P* ≤ 0.05 and a fold change ratio ≥1.5.

### Identification of apical surface proteins, prediction of interactions and functional analysis

Proteins were considered as apical surface proteins if they were (I) not predicted to be intracellular and (II) previously identified in human cilia or (III) annotated with the Gene Ontology Cellular Component (GO CC) plasma membrane (GO:0005886) in the Metascape membership tool (Zhou *et al.* 2019) or (IV) predicted to be transmembrane. The subcellular location of the proteins was further verified using UniProt, and proteins located at cell junctions or in the cytoplasm were excluded. Prediction of membrane lipid-binding proteins was performed using the online MBPPred tool, which identifies membrane-binding domains (http://aias.biol.uoa.gr/MBPpred/index.php) (Nastou *et al.* 2016). Prediction of interactions with the sperm-coating BSPA1/2, BSPA3 or BSP30kD and DEFB126 proteins was performed using the ProteinPrompt tool with random forest computation method, mammalian database and a minimum interaction score of 0.4 (https://proteinformatics.uni-leipzig.de/ProteinPrompt/) (Canzler *et al.* 2022).

Functional enrichment analysis of GO Molecular Function (GO MF) was carried out in Metascape using the *Homo sapiens* database as the background (Zhou *et al.* 2019). The HUGO Gene Nomenclature Committee Comparison of Orthology Predictions search tool was used to retrieve the human ortholog gene names (https://www.genenames.org/).

### Immunodetection and localization of catalase (CAT), oviduct glycoprotein 1 (OVGP1) and peroxiredoxin-6 (PRDX6)

Histological sections (3 μm) were prepared from each ampulla and isthmus sample. After deparaffinization and rehydration, antigen retrieval was performed by microwaving in 10 mmol/L sodium citrate buffer pH 6.0 for 8 min. Endogenous peroxidase activity was blocked with 3% H_2_O_2_ in methanol for 10 min. To minimize nonspecific binding, sections were incubated in blocking buffer containing 5% BSA (Carl Roth, Germany) and 2% horse serum (MP-7402; Vector Laboratories Inc, USA) for 1 h. Afterward, the sections were incubated with the primary rabbit polyclonal antibodies diluted in blocking buffer overnight at 4°C: anti-CAT (1.1 μg/mL; 21260-1-AP, Proteintech, Germany), anti-OVGP1 (2.5 μg/mL; ab118590, Abcam, UK), and anti-PRDX6 (1.25 μg/mL; ABIN2783305, Antibodiesonline, USA). Negative controls were conducted by replacing the primary antibody with a rabbit isotype control polyclonal antibody (N1001, NSJ Bioreagents, USA). After primary antibody labeling, sections were incubated with peroxidase-conjugated anti-rabbit IgG (MP-7401, ImmPRESS® HRP Horse Anti-Rabbit IgG Polymer Detection Kit, Vector Laboratories, USA) for 1 h at room temperature. Immunodetection was visualized using diaminobenzidine substrate chromogen solution (DAB+, K3468, Agilent Dako, USA). The slides were counterstained with hematoxylin. Microscopical pictures were taken using a Zeiss Axioplan microscope equipped with an Axiocam 305 color camera and ZEN software 3.10 (Carl Zeiss AG, Germany).

## Results

### Proteins identified in the apical and basal microsamples of the oviduct epithelium

A total of 505 proteins were identified and quantified in the microsamples derived from the apical part of the epithelium (Supplementary Table S1) and 512 in those collected from the basal part (Supplementary Table S2). The majority of proteins were shared between both parts of the epithelium: only four proteins with an average of more than two precursor ion intensities were specifically identified in the apical and 11 in the basal compartment (Table 1).

**Table 1:** Proteins identified either exclusively in the apical or in the basal microsamples. Proteins were considered as identified with an average of more than two ion precursor intensities (quantitative value).

Protein name	Total mean ion precursor intensities
Proteins exclusively identified in the apical microsamples	
Histone H2B (H2BC11)	12.7
H1.4 linker histone, cluster member (H1-4)	7.2
ADP/ATP translocase 2 (SLC25A5)	2.7
Poly(rC) binding protein 2 (PCBP2)	2.1
Proteins exclusively identified in the basal microsamples	
40S ribosomal protein S13 (RPS13)	45.1
ATP synthase subunit f, mitochondrial (ATP5MF)	21.4
Synaptotagmin binding cytoplasmic RNA interacting protein (SYNCRIP)	18.3
60S ribosomal protein L15 (RPL15)	15.9
Histone H3.1; H3.3C-like (H31; H3CL)	11.6
60S ribosomal protein L9 (RPL9)	11.1
Histone deacetylase 1 (HDAC1)	11.1
Serine/threonine-protein phosphatase PP1-beta catalytic subunit (PPP1CB)	8.4
60S ribosomal protein L17 (RPL17)	8.4
Annexin A11 (ANXA11)	7.8
Nucleoside-triphosphatase, cancer-related (NTPCR)	6.5

Among proteins of both the apical and basal microsamples, 21% (106/505 and 106/512, respectively) were previously identified in human cilia, 10% (53/505 and 53/512, respectively) were predicted to be transmembrane, 59–60% (297/505 and 306/512, respectively) intracellular and 38% (194/505 and 192/512, respectively) secreted (Supplementary Tables S1 and S2).

### Differentially abundant proteins between oviduct regions and peri-ovulatory stages in the apical and basal part of the oviduct epithelium

**Figure 3:**
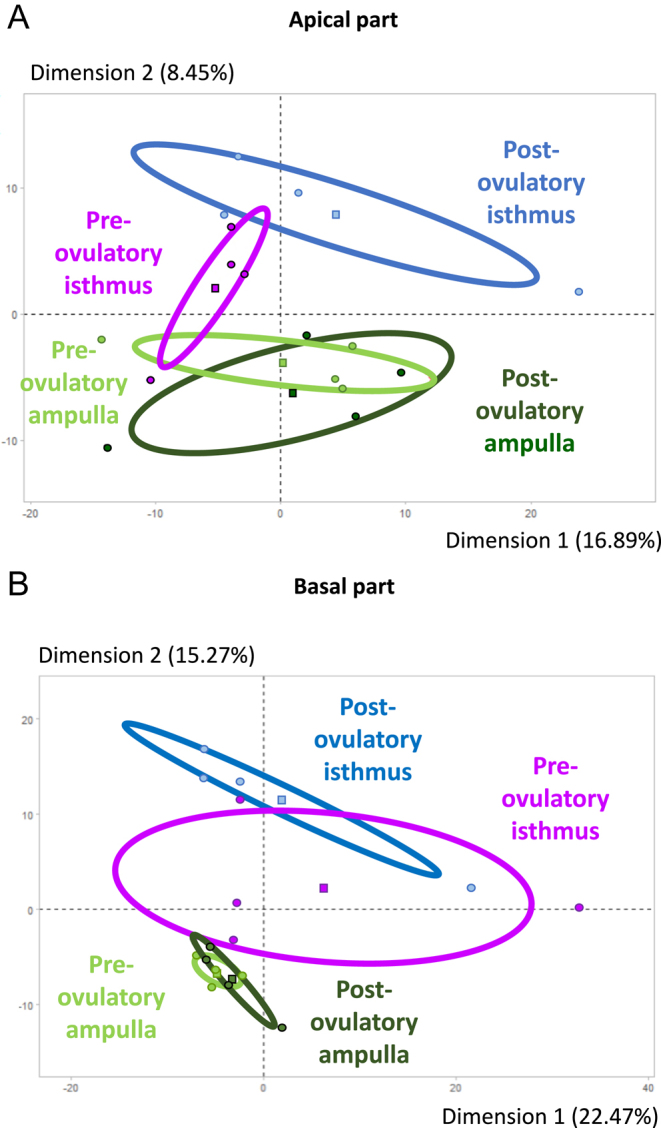
Principal component analysis of the 16 microdissected samples from the (A) apical and (B) basal part of the bovine oviduct epithelium. Principal component analysis was carried out on all samples using log2 quantitative values and RStudio software. Each filled circle represents one sample. Squares represent the mean in each condition. Confidence ellipses included 95% of all samples. The percentages of each dimension represent the total variance of data.

**Figure 4:**
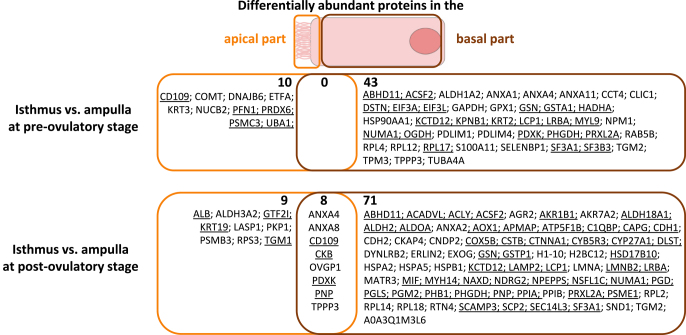
Differential protein abundance between regions (isthmus and ampulla) in the apical and basal part of the bovine oviduct epithelium. Proteins were considered differentially abundant after paired t-tests on log2 precursor ion intensities with a *P*-value ≤0.05 and fold change ratio ≥1.5. Underlined protein symbols = overabundant in isthmus compared to ampulla.

**Figure 5:**
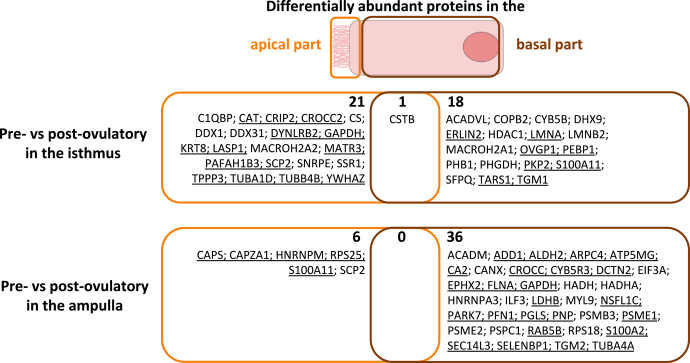
Differential protein abundance between cycle stages (pre- and post-ovulatory) in the apical and basal part of the bovine oviduct epithelium. Proteins were considered differentially abundant after unpaired t-tests on log2 precursor ion intensities with a *P*-value ≤0.05 and fold change ratio ≥1.5. Underlined protein symbols = overabundant at pre-ovulatory compared to post-ovulatory stage.

In both epithelium parts, PCA of all samples showed a segregation of samples between ampulla and isthmus (Fig. 3). In addition, the PCA also showed a segregation between pre- and post-ovulatory samples in the isthmus, especially in the apical part of the epithelium (Fig. 3A). In the apical part of the epithelium, pairwise comparison revealed ten and 17 DAPs between the ampulla and isthmus at pre- and post-ov stages, respectively. CD109 molecule (CD109) and peroxiredoxin-6 (PRDX6) were among the most overabundant DAPs in pre-ov isthmus compared to pre-ov ampulla. Furthermore, six and 22 DAPs were detected between pre- and post-ov stage in ampulla and isthmus, respectively, including catalase (CAT) as one of the most overabundant DAPs in pre-ov isthmus compared to post-ov isthmus (Figs 4 and 5; Supplementary Table S3). In the basal part, 43 and 79 DAPs were detected between regions at pre-ov and post-ov stages, respectively. Furthermore, 19 and 36 DAPs were evidenced between stages in the isthmus and ampulla, respectively, including oviduct glycoprotein 1 (OVGP1) as one of the most overabundant DAPs in pre-ov isthmus compared to post-ov isthmus (Figs 4 and 5; Supplementary Table S4).

Although the majority of proteins were shared between the apical and basal part of the epithelium, very few DAPs were shared between both cell compartments. When considering the effect of the oviduct region (isthmus/ampulla) on protein abundance, only eight out of 141 DAPs followed the same pattern of regulation in both parts of the epithelium: pyridoxal kinase (PDXK), purine nucleoside phosphorylase (PNP), creatine kinase B-type (CKB) and CD109 molecule (CD109) were more abundant in the isthmus than in ampulla, while annexin A4 and A8 (ANXA4, ANXA8), tubulin polymerization-promoting protein family member 3 (TPPP3) and oviduct glycoprotein 1 (OVGP1) were more abundant in the ampulla than in the isthmus at the post-ov stage (Fig. 4). Considering the effect of cycle stage on protein abundance, out of 79 DAPs, only cystatin-B (CSTB) was more abundant in both parts of the isthmic epithelium at post-ov than pre-ov (Fig. 5).

### Immunodetection and localization of CAT, PRDX6 and OVGP1

**Figure 6:**
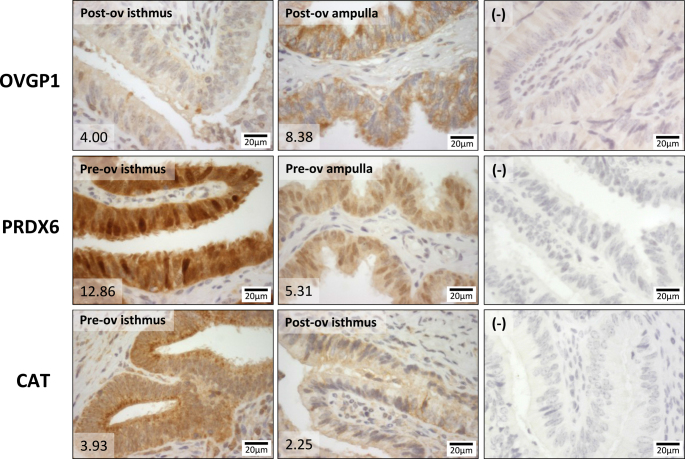
Immunohistochemical detection of oviduct glycoprotein 1 (OVGP1), peroxiredoxin 6 (PRDX6) and catalase (CAT) in bovine oviduct tissue. Representative pictures of OVGP1 (post-ovulatory isthmus vs post-ovulatory ampulla), PRDX6 (pre-ovulatory isthmus vs pre-ovulatory ampulla) and CAT (pre-ovulatory isthmus vs post-ov isthmus) localization and staining intensity in bovine oviduct tissue. Numbers in the lower left corners represent the precursor ion intensities measured by nanoLC-MS/MS in microsamples of the apical epithelium part of the same samples. Scale bar: 20 µm. Counterstain: hematoxylin.

Proteins (CAT, PRDX6 and OVGP1) with a high differential abundance between regions and stages in the apical part of the epithelium were selected for qualitative validation and immunolocalization. All proteins were detected in the apical part and basal part of the epithelium. For the three proteins, the staining intensities in the apical cell compartment followed the quantification pattern measured by nanoLC-MS/MS (Fig. 6).

### Potential candidates for sperm binding in the reservoir

**Figure 7:**
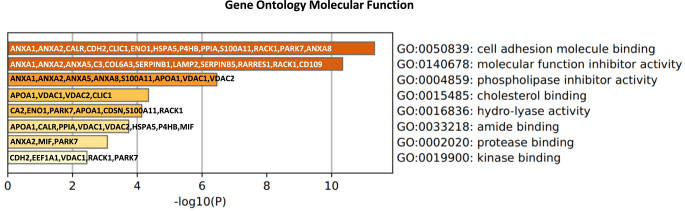
Functional enrichment analysis of 41 predicted apical surface proteins in the bovine oviduct epithelium. Functional analysis was performed using the Metascape tool online (Zhou *et al.* 2019). Gene symbols were used as input and the *Homo sapiens* database as background.

To identify candidates for sperm binding to OECs, we evaluated those apical proteins that are potentially localized at the surface of the epithelium. A total of 41 proteins were considered to be apical surface proteins (see Table 2). Based on GO MF, these proteins were involved among others in: cell adhesion molecule binding, including ANXA1, ANXA2, ANXA8 and HSPA5; phospholipase inhibitor activity, including ANXA1, ANXA2, ANXA5, ANXA8, apolipoprotein A-I (APOA1) and voltage-dependent anion-selective channel protein (VDAC) 1 and 2; and cholesterol binding, including APOA1, VDAC1 and VDAC2 (Fig. 7).

**Table 2:** List of the 41 predicted apical surface proteins. Localization of proteins in cilia was assessed using the Human Protein Atlas entries for ‘cilia’ or ‘cilium’ (Uhlén *et al.* 2015) and lists of proteins previously identified in human airway cilia (Blackburn *et al.* 2017). Potential membrane localization of proteins was determined using the membership tool of Metascape (Zhou *et al.* 2019) for plasma membrane proteins and TMHMM 2.0 for transmembrane proteins (Krogh *et al.* 2001). Prediction of interaction with the membrane was performed using MBPPred (Nastou *et al.* 2016) and with the bovine seminal plasma proteins (BSPs) and the defensin beta 126 (DEFB126) using ProteinPrompt (Canzler *et al.* 2022). Sperm-interacting proteins were identified using lists of proteins previously identified as interacting with spermatozoa in the bovine oviduct fluid (Mahé *et al.* 2023) or on bovine oviduct cells (Boilard *et al.* 2004, Ignotz *et al.* 2007).

Protein name	Gene symbol	Localization in membrane and/or cilia	Predicted interaction with the membrane, BSPs and/or DEFB126	Sperm-interacting proteins in the bovine oviduct
Anterior gradient 2, protein disulfide isomerase family member	*AGR2*	Previously identified in human cilia		
Annexin A1	*ANXA1*	Previously identified in human cilia and annotated as plasma membrane protein	Predicted to interact with the membrane	Previously identified as sperm-interacting protein in the fluid and on cells
Annexin A2	*ANXA2*	Previously identified in human cilia and annotated as plasma membrane protein	Predicted to interact with the BSPA1,2, BSP30kD, DEFB126, and membrane	Previously identified as sperm-interacting protein in the fluid and on cells
Annexin A5	*ANXA5*	Previously identified in human cilia and annotated as plasma membrane protein	Predicted to interact with the BSPA3, BSP30kD and membrane	Previously identified as sperm-interacting protein in the fluid and on cells
Annexin A8	*ANXA8*	Annotated as plasma membrane protein	Predicted to interact with the BSP30kD, and membrane	Previously identified as sperm-interacting protein in the fluid and on cells
Apolipoprotein A-I	*APOA1*	Annotated as plasma membrane protein	Predicted to interact with the BSP30kD and DEFB126	
Complement C3	*C3*	Annotated as plasma membrane protein		
Carbonic anhydrase 2	*CA2*	Annotated as plasma membrane protein		
Calreticulin	*CALR*	Annotated as plasma membrane protein	Predicted to interact with the BSPA1,2,3, BSP30kD and DEFB126	
CD109 molecule	*CD109*	Predicted as transmembrane		Previously identified as sperm-interacting protein in the fluid
Cadherin 2	*CDH2*	Predicted as transmembrane	Predicted to interact with the BSP30kD	
Corneodesmosin	*CDSN*	Predicted as transmembrane		
Chloride intracellular channel protein 1	*CLIC1*	Previously identified in human cilia and annotated as plasma membrane protein	Predicted to interact with the BSP30kD and DEFB126	Previously identified as sperm-interacting protein in the fluid
Collagen type VI alpha 3 chain	*COL6A3*	Annotated as plasma membrane protein		
Catechol O-methyltransferase	*COMT*	Predicted as transmembrane		
Coatomer subunit beta	*COPB1*	Predicted as transmembrane		
Cytochrome P450, family 4, subfamily B, polypeptide 1	*CYP4B1*	Previously identified in human cilia and predicted as transmembrane		
L-xylulose reductase	*DCXR*	Annotated as plasma membrane protein		
Elongation factor 1-alpha 1	*EEF1A1*	Previously identified in human cilia and annotated as plasma membrane protein	Predicted to interact with the BSPA1,2,3 and BSP30kD	Previously identified as sperm-interacting protein in the fluid
Alpha-enolase	*ENO1*	Previously identified in human cilia and annotated as plasma membrane protein		
Endoplasmic reticulum chaperone BiP	*HSPA5*	Previously identified in human cilia and annotated as plasma membrane protein	Predicted to interact with the BSP30kD	Previously identified as sperm-interacting protein in the fluid and on cells
Lysosomal associated membrane protein 2	*LAMP2*	Predicted as transmembrane		
Macrophage migration inhibitory factor	*MIF*	Annotated as plasma membrane protein		
Oviduct glycoprotein 1	*OVGP1*	Previously identified in human cilia		Previously identified as sperm-interacting protein in the fluid
Protein disulfide-isomerase	*P4HB*	Annotated as plasma membrane protein		
Parkinson disease protein 7	*PARK7*	Previously identified in human cilia and annotated as plasma membrane protein	Predicted to interact with the DEFB126	
Prohibitin 1	*PHB1*	Predicted as transmembrane	Predicted to interact with the BSPA1,2,3 and BSP30kD	
Plexin B2	*PLXNB2*	Annotated as plasma membrane protein		
Peptidyl-prolyl cis–trans isomerase A	*PPIA*	Previously identified in human cilia	Predicted to interact with the BSPA1,2,3, BSP30kD and DEFB126	Previously identified as sperm-interacting protein in the fluid
Peroxiredoxin-like 2A	*PRXL2A*	Predicted as transmembrane		
Ras-related protein Rab-11A	*RAB11A*	Annotated as plasma membrane protein	Predicted to interact with the BSPA1,2,3 and BSP30kD	
Receptor of activated protein C kinase 1	*RACK1*	Previously identified in human cilia and annotated as plasma membrane protein	Predicted to interact with the BSPA1,2,3, BSP30kD and DEFB126	
Retinoic acid receptor responder (Tazarotene induced) 1	*RARRES1*	Previously identified in human cilia		
Protein S100	*S100A11*	Previously identified in human cilia		
Leukocyte elastase inhibitor	*SERPINB1*	Previously identified in human cilia		
Serpin B5	*SERPINB5*	Annotated as plasma membrane protein		
Tumor associated calcium signal transducer 2	*TACSTD2*	Predicted as transmembrane		
Transmembrane emp24 domain-containing protein 10	*TMED10*	Predicted as transmembrane		
Voltage-dependent anion-selective channel protein 1	*VDAC1*	Previously identified in human cilia and annotated as plasma membrane protein	Predicted to interact with the BSPA1,2,3, BSP30kD and DEFB126	
Voltage-dependent anion-selective channel protein 2	*VDAC2*	Previously identified in human cilia	Predicted to interact with the BSPA1,2, BSP30kD and DEFB126	
BOLA class I histocompatibility antigen, alpha chain BL3-7		Predicted as transmembrane		

**Figure 8:**
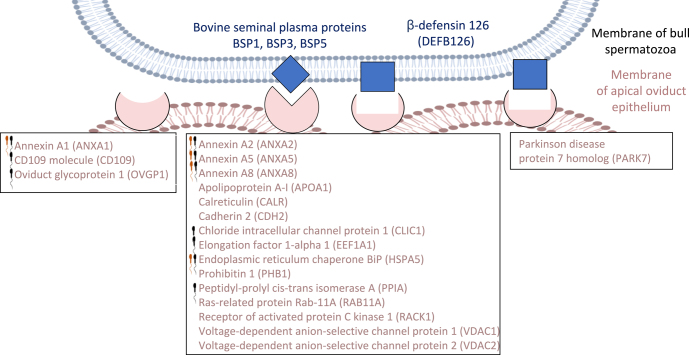
Potential candidates for sperm binding to oviduct epithelial cells in the reservoir. Black spermatozoa = proteins previously identified as sperm-interacting proteins in the bovine oviduct fluid (Mahé *et al.* 2023); orange spermatozoa = proteins previously identified as sperm-interacting proteins at the surface of bovine oviduct epithelial cells (Boilard *et al.* 2004, Ignotz *et al.* 2007).

Among these 41 surface proteins, ten were previously identified as sperm-interacting proteins in the oviduct fluid (Mahé *et al.* 2023) or on the surface of oviduct epithelial cells in cattle (Boilard *et al.* 2004, Ignotz *et al.* 2007) such as ANXA1, ANXA2, ANXA5 and ANXA8, HSPA5 and OVGP1. Prediction of interactions revealed four proteins interacting with membrane lipids (ANXA1, 2, 5 and 8), 15 proteins interacting with PDC-109 (BSP1), BSP3 or BSP5 and nine with DEFB126, including ANXA2, APOA1, VDAC1 and VDAC2. A total of 19 potential candidates for sperm binding to OECs were identified and summarized in Fig. 8.

## Discussion

By combining laser capture microdissection and nanoLC-MS/MS, this study reports a spatiotemporal profiling of the bovine oviduct epithelial proteome at the subcellular level using paraformaldehyde-fixed, paraffin-embedded tissue samples. We identified 505 proteins in the apical and 512 in the basal part of the epithelium, representing a comprehensive proteome of both epithelial cell compartments of the bovine isthmus and ampulla. From these proteins, 41 were considered to be apical surface proteins. By combining comparative analyses with previously identified sperm-interacting proteins and *in silico* predictive analysis of molecular interactions, we proposed 19 candidates for sperm-isthmus cell interactions in the reservoir.

To our knowledge, this is the first spatiotemporal proteomic study separating the apical and basal parts of the mammalian oviduct epithelium. Recent transcriptomic studies in the mammalian oviduct have mainly focused on the whole tissue, e.g. in pig (Martyniak *et al.* 2018, Jang *et al.* 2022) and sheep (López-Úbeda *et al.* 2017, Chen *et al.* 2023), or on a mixture of epithelial and stromal cells in cattle (Locatelli *et al.* 2019, Stamperna *et al.* 2022). In addition, studies in rabbits and pigs analyzed the apical proteome of the oviduct epithelium after protein biotinylation and LC-MS/MS but without differentiating the ampulla from the isthmus and with less in-depth analysis, identifying 280 proteins (Sostaric *et al.* 2006, Artemenko *et al.* 2015).

In both subcellular compartments, the region (isthmus/ampulla) was the major source of variation in protein abundance before the cycle stage, as indicated by the PCA and the number of DAPs identified. This is consistent with our previous proteomic study of the bovine oviduct fluid in which the region had the greatest effect on protein abundance compared to the side relative to ovulation (ipsi- vs contralateral to ovulation) and cycle stage (pre- vs post-ov) (Mahé *et al.* 2022). In addition, significant differences in mRNA abundance between the isthmus and ampulla have been previously demonstrated (Maillo *et al.* 2016, Locatelli *et al.* 2019, Mahé *et al.* 2022, Martyniak *et al.* 2023). These differences may reflect the specific functions of each region during the periconceptional period. Before ovulation, the ciliated cells of the isthmic epithelium, in particular, interact with spermatozoa to prolong their lifespan (Sostaric *et al.* 2008, Kölle 2022). After ovulation, cilia of the ampullary epithelium guide the oocyte–cumulus complex toward the ampulla-isthmus junction. In case of a successful fertilization, both ampulla and isthmus secretions support the embryo in its early development and ciliary beating in the isthmus guides the embryo toward the uterus (Li & Winuthayanon 2017). All the above-mentioned dynamic processes are stage-specific and occur at the apical surface of the epithelium. Accordingly, our results suggest that protein abundance is regulated by the cycle stage, especially in the apical part of the epithelium. The PCA results showed that the samples segregated according to peri-ovulatory stage. This segregation was more pronounced in the isthmus than in the ampulla and also stronger in the apical part of the epithelium than in the basal part. Although 98% of the identified proteins were present in both cell compartments, they shared very few DAPs (<2%) according to region and cycle stage, also indicating specificity of regulation in each part of the epithelium.

Interestingly, two of the most strongly regulated proteins in the apical part of the pre-ovulatory isthmus are enzymes involved in the regulation of sperm capacitation and protection of mammalian spermatozoa against oxidative stress: CAT and PRDX6 (O’Flaherty 2018, Aitken & Drevet 2020). Immunohistochemical detection of PRDX6 not only confirmed its stronger expression in the pre-ovulatory isthmus compared to pre-ovulatory ampulla but revealed its localization in the cilia of the pre-ovulatory isthmic epithelium. This hints toward a specific role of this enzyme in the fine-tuned control of reactive oxygen species within the functional sperm reservoir.

Direct contact between spermatozoa and the plasma membrane of OECs is probably required *in vivo* to maintain sperm viability and facilitate the acquisition of fertilizing ability (Smith & Nothnick 1997, Boilard *et al.* 2004). After *in silico* analysis, we proposed 41 apical surface proteins. Accordingly, these proteins were significantly enriched in GO MF terms related to binding such as ‘cell adhesion molecule binding’ and ‘cholesterol binding’. Among these surface proteins, 19 were considered as solid candidates for sperm binding to the apical oviduct, including ten previously identified as sperm-interacting proteins in the bovine oviduct fluid (Mahé *et al.* 2023) and five at the surface of OECs (Boilard *et al.* 2004, Ignotz *et al.* 2007). Some of these candidates have reported roles in sperm functions. In buffalo, purified OVGP1 from the oviduct fluid bound to spermatozoa and increased their viability and fertilizing ability compared to control without OVPG1 (Choudhary *et al.* 2017). In bovine oviduct, HSPA5 was identified on the apical plasma membranes of OECs as a sperm receptor involved in maintaining sperm viability and acrosome integrity (Boilard *et al.* 2004). In addition, seven of these candidates, ANXA1, ANXA2, ANXA5, ANXA8, APOA1, VDAC1 and VDAC2, were enriched in the GO MF ‘phospholipase inhibitor activity’. Phospholipases are a family of enzymes that hydrolyze membrane glycerophospholipids. In mice, two types of phospholipase A2 are involved in sperm flagellar beating and in the activation of the acrosome reaction (Arnoult *et al.* 2012). We hypothesize that these seven proteins could maintain motility and prevent premature acrosome reaction of spermatozoa during their time in the reservoir.

The 19 candidates proposed by our analysis could interact with spermatozoa through protein–protein interactions. BSPs are a superfamily of seminal plasma-derived proteins that coat spermatozoa during ejaculation and have been identified as OEC receptors in cattle (Gwathmey *et al.* 2006). After demonstrating that BSP-free spermatozoa were still able to bind to OECs *in vitro*, epididymal-derived proteins such as DEFB126 were identified to act as OEC receptors on bovine (Lyons *et al.* 2018) and macaque (Tollner *et al.* 2008) sperm. Of the 19 candidates suggested here, 15 were predicted to interact with the seminal plasma proteins BSP1, BSP3 or BSP5 and nine with DEFB126, including ANXA2, APOA1, VDAC1 and VDAC2. In cattle, ANXA2 has already been identified as a binding partner of BSPs (Ignotz *et al.* 2007), but this is the first report of its possible interactions with DEFB126. In addition, there is already experimental evidence for the interaction of APOA1 and BSPs, as affinity chromatography purification of BSP-binding proteins in human plasma revealed APOA1 as the major binding partner (Manjunath *et al.* 1989). APOA1 has also been identified in the oviduct fluid of cyclic cows (Mahé *et al.* 2022) and acts as a cholesterol acceptor during *in vitro* induced sperm capacitation in humans (Langlais *et al.* 1988). Thus, it is possible that sperm binding to APOA1 via BSPs is involved in the acquisition of fertilizing ability in sperm residing in the oviduct reservoir. Other proteins such as VDAC1 and VDAC2 have previously been identified as sperm-interacting proteins in ovine cervical mucus fluid (Soleilhavoup 2015). In cattle, incubation of spermatozoa with antibodies against VDAC1 and VDAC2 increased the proportion of spermatozoa without an acrosome compared to the control group without antibodies (Triphan *et al.* 2007). In the reservoir, sperm interaction with VDAC1 and VDAC2 may therefore play a role in maintaining acrosome integrity.

Glycerophospholipids and cholesterol are major components of the bull sperm plasma membrane (Gautier & Aurich 2022). Binding of spermatozoa to the apical oviduct epithelium could also be mediated by protein-lipid interactions. Accordingly, VDAC proteins have been reported to interact with anionic lipid-like cardiolipin or phosphatidylglycerol (Betaneli *et al.* 2012). Annexins are another family of proteins that bind with high affinity to various membrane phospholipids, including phosphatidylserine, in a calcium- or pH-dependent manner (Lizarbe *et al.* 2013). ANXA1, A2, A5, and A8 were predicted to have a membrane binding domain and could directly bind to sperm membrane bilayers. In pigs, ANXA5, located on the ciliary surface of OECs, interacts with the heads of capacitated spermatozoa via phosphatidylserine binding (Schmaltz *et al.* 2025).

## Conclusion

This work provides the first comprehensive analysis of the proteins abundant in the apical and basal part of the bovine oviduct epithelium before and after ovulation. Protein abundance in both subcellular compartments was differentially regulated according to the oviduct region and time relative to ovulation, offering new insights into the mechanisms underlying fertilization success and new protein candidates for oviduct–sperm interactions. Further functional studies are needed to elucidate their effects on gamete and early embryo survival and quality.

## Supplementary materials









## Declaration of interest

The authors declare that there is no conflict of interest that could be perceived as prejudicing the impartiality of the research reported.

## Funding

This work was funded by INRAE, the National Research Agency (ANR-18-CE92-0049), the Interdisciplinary access call to Structural Biology, Biological Imaging and Proteomics IBiSA facilities 2022 and the German Research Foundation (DFG Scho1231/7-1). This work was also supported by structural grants from Biogenouest, Infrastructures en Biologie Santé et Agronomie (IBiSA) and the Conseil Régional de Bretagne awarded to CP.

## Author contribution statement

MSD, PM and JS designed and supervised the study. MSD and JS acquired funding and wrote the manuscript. CM analyzed the proteomic data and wrote the manuscript. AMM and EA performed the laser microdissection. MSD and KR collected and prepared oviduct tissues for laser microdissection. RL, EC and CP did the MS/MS analysis. All authors have read and agreed to the published version of the manuscript.
